# MOCR-DB: The Multi-Omics Causal Resource Database for Genetic Correlation, Causal Inference, and Functional Interpretation

**DOI:** 10.1007/s43657-026-00338-w

**Published:** 2026-08-28

**Authors:** Hongwei Chen, Bingjie Fan, Huiyao Chen, Yongkun Chen, Yuelong Shu, Haoyang Zhang

**Affiliations:** 1 School of Public Health (Shenzhen), Shenzhen Key Laboratory of Pathogenic Microbes and Biosafety, Shenzhen Campus of Sun Yat-sen University, Shenzhen, 518107 Guangdong China; 2 School of Public Health (Shenzhen), Sun Yat-sen University, Guangzhou, 510275 Guangdong China; 3 Department of CICU, Guangzhou Women and Children’s Medical Center, Guangzhou Medical University, Guangzhou, 510623 Guangdong China; 4 Center for Molecular Medicine, Children’s Hospital of Fudan University, Shanghai, 201102 China; 5 Guangdong Provincial Key Laboratory of Infection Immunity and Inflammation, School of Basic Medical Sciences, Shenzhen University Medical School, Shenzhen University, Shenzhen, 518060 China; 6 Key Laboratory of Pathogen Infection Prevention and Control (MOE), State Key Laboratory of Respiratory Health and Multimorbidity, National Institute of Pathogen Biology, Chinese Academy of Medical Sciences & Peking Union Medical College, Beijing, 102629 China; 7 Department of Experimental Medical Science, Lund University, Lund, 22184 Sweden

**Keywords:** Genome-wide association study, Quantitative trait locus, Genetic correlation, Causal inference, Functional genomics, Database

## Abstract

Genome-wide association studies (GWAS) have revealed extensive polygenic signals and overlapping genetic architectures across human traits, creating a need for resources that connect trait-level genetic relationships with gene-level functional evidence. Here, we developed the Multi-Omics Causal Resource Database (MOCR-DB), an interactive platform that integrates large-scale GWAS summary statistics from UK Biobank, FinnGen, and the COVID-19 Host Genetics Initiative with molecular quantitative trait locus (QTL) datasets. In total, 613 traits with significant heritability were retained and harmonized using the Unified Medical Language System. MOCR-DB integrates phenotype-to-phenotype analyses, including genetic correlation and Mendelian randomization, with phenotype-to-gene analyses based on QTL-informed summary-data-based Mendelian randomization analysis (SMR) within a single searchable and interactive framework. The platform supports exploration of cross-trait genetic correlations, putative causal relationships, and candidate functional gene associations. An AI-assisted module provides concise plain-language summaries to help contextualize statistical findings. As a case study, we examined obesity and COVID-19 severity, where genetically predicted obesity showed a stronger association with critical COVID-19 and lung eQTL-based SMR analyses revealed distinct immune- and neuronal-related molecular patterns across severity groups. MOCR-DB thus provides a unified and accessible resource for investigating shared genetic architectures and prioritized functional gene candidates across complex traits, supporting the generation of reproducible and biologically interpretable hypotheses. The database is publicly available at https://chenhongwei.net/public/MOCRdb/.

**Graphical Abstract:**

**Data resources, analytical framework, and interpretation in MOCR-DB **The Multi-Omics Causal Resource Database (MOCR-DB) integrates large-scale GWAS summary statistics and molecular QTL datasets to provide a unified framework for genetic correlation, causal inference, and functional mediation. Data resources include GWAS summary statistics from UK Biobank, FinnGen, and the COVID-19 Host Genetics Initiative, together with 53 xQTL datasets across 49 tissues (eQTL, mQTL, sQTL, and caQTL). The analytical framework combines linkage disequilibrium score regression (LDSC) for estimating heritability and cross-trait genetic correlation, Mendelian randomization (MR) to infer potential causal relationships between traits, and summary-data-based Mendelian randomization (SMR) to identify tissue-specific functional genes. Results are presented through interactive genetic network searches that link diseases, biomarkers, lifestyle factors, and molecular traits via correlation, causality, and functional annotation. An AI-assisted module further facilitates causal and functional interpretation by summarizing complex results from LDSC, MR, and SMR analyses into accessible biological insights. Together, MOCR-DB provides systematic exploration of shared genetic architectures and functional mediators across complex human traits.

**Figure:**
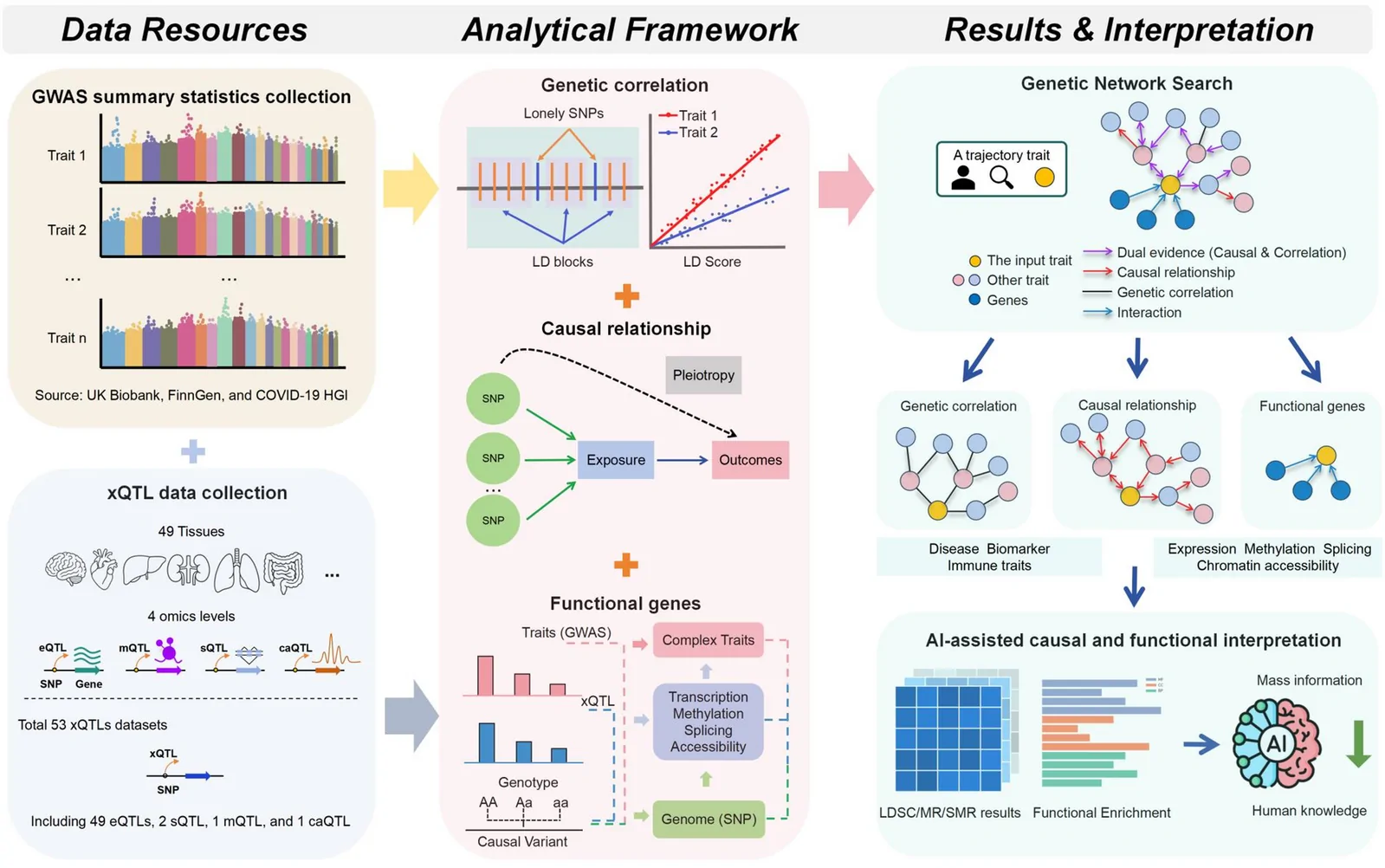

**Supplementary Information:**

The online version contains supplementary material available at https://doi.org/10.1007/s43657-026-00338-w.

## Introduction

In recent years, genome-wide association studies (GWAS) with increasingly large sample sizes have greatly advanced the understanding of complex human traits and diseases (Boyle et al. 2017; Tam et al. 2019; Waldron 2016). A consistent finding is that many phenotypes share overlapping genetic architecture (Bulik-Sullivan et al. 2015a; Grotzinger et al. 2019; Pickrell et al. 2016), underscoring the value of studying relationships across traits to reveal common mechanisms and potential therapeutic targets (Watanabe et al. 2019). Beyond identifying risk loci for single traits, cross-trait analyses of shared genetics can highlight pleiotropic pathways, inform drug repurposing opportunities, and improve the understanding of comorbidity patterns across diverse disease domains (Nelson et al. 2015).

Several statistical approaches have been developed to support such investigations, including methods that quantify genetic correlations, infer potential causal directions, and link associations to molecular regulation (Bulik-Sullivan et al. 2015b). Together, they provide a layered perspective connecting genome-wide correlation with functional mechanisms (Hemani et al. 2018). A variety of resources have been built upon these methods, yet they differ substantially in scope and analytical focus. For example, DMRdb (Zheng et al. 2025) concentrates on trait-level Mendelian randomization, whereas the recently developed MendelR package (Ke et al. 2025) offers a more comprehensive analytical framework that integrates GWAS with multiple molecular QTL datasets and automates key steps of MR analysis, including data harmonization and instrument selection. Meanwhile, the SMR-Portal (Guo et al. 2025; Zhu et al. 2016) is dedicated to linking GWAS with multiple molecular QTL layers, including gene expression (eQTL) (Consortium 2020), splicing (sQTL) (Qi et al. 2022), methylation (mQTL) (Oliva et al. 2023), and chromatin accessibility (caQTL) (Bryois et al. 2018). A few integrative tools, such as EpiGraphDB (Liu et al. 2021) or FUMA (Watanabe et al. 2017), incorporate functional annotations or network representations, yet their capacity to connect phenotype-to-phenotype relationships with phenotype-to-gene functional associations is still limited, and the coverage of infectious and molecular traits remains relatively narrow.

Based on these considerations, we developed the Multi-Omics Causal Resource Database (MOCR-DB). MOCR-DB was designed to bridge phenotype-to-phenotype analyses, including genetic correlation and Mendelian randomization, with phenotype-to-gene functional analyses based on molecular QTL-informed SMR. Rather than presenting these methods as separate analytical layers, MOCR-DB provides a unified and interactive framework for exploring heritability, cross-trait genetic relationships, putative causal directions, and candidate functional gene associations across hundreds of traits. The database covers a broad spectrum of infectious and non-infectious traits and molecular phenotypes, and it supports network-based visualization, functional annotation, and AI-assisted interpretation. To our knowledge, MOCR-DB provides a practical large-scale resource that links trait-level relationships with gene-level evidence, supporting the exploration of shared genetic mechanisms across complex human traits.

## Materials and Methods

### Database Overview

This study aimed to construct the MOCR-DB, an interactive platform designed to explore genetic relationships across complex traits and diseases. MOCR-DB integrates large-scale GWAS summary statistics with multiple downstream analytical pipelines, including single-trait SNP-based heritability estimation, cross-trait genetic correlation, causal effect estimation, and functional annotation. Together, these analyses provide an integrated approach for linking genetic associations with downstream functional annotations.

### Data Sources and Processing

MOCR-DB integrates GWAS summary statistics from multiple large-scale resources to provide comprehensive coverage of complex traits and diseases. The primary datasets were obtained from UK Biobank (UKB) (Jiang et al. 2019; Sudlow et al. 2015), FinnGen (Heyne et al. 2023; Kurki et al. 2023), and the COVID-19 HGI (release 7, hg38) (Initiative 2023), which represent large European cohorts with extensive phenotypic information. For continuous traits, all available GWAS results were included, whereas traits with insufficient sample sizes or incomplete summary statistics were excluded. For binary traits, only those with case proportions above 1% were retained to ensure sufficient statistical power. Within the COVID-19 HGI data, two phenotypes were selected for integration: very severe respiratory confirmed COVID-19 (A2) and hospitalized COVID-19 (B2). All summary statistics were obtained from publicly accessible repositories, including FinnGen (https://www.finngen.fi/), the GCTA data portal (https://yanglab.westlake.edu.cn/software/gcta/#DataResource) providing UK Biobank GWAS and QTL resources, and the COVID-19 HGI website (https://www.covid19hg.org/results/r7/).

To harmonize terminology across different cohorts, the Unified Medical Language System (UMLS) (Bodenreider 2004) was applied (e.g., unifying terms such as "diabetes" and "diabetes mellitus", "hypertension" and "high blood pressure", or "influenza" and "flu"). For consistency, all GWAS summary statistics were aligned to the 1000 Genomes Project (1KGP) reference panel, and SNPs located within the Major Histocompatibility Complex (MHC) region were excluded owing to the complex LD structure (da Silva et al. 2013). The retained fields for MR analysis included SNP identifiers, A1 (effect allele), A2 (other allele), FRQ (effect allele frequency), BETA, SE, P, and N (sample size), ensuring compatibility with downstream causal inference.

With these harmonized datasets established, we next applied linkage disequilibrium score regression (LDSC) as the first analytical step to evaluate the polygenic architecture of traits included in MOCR-DB (Bulik-Sullivan et al. 2015a, b). LD scores were pre-computed from the 489 European samples in the 1000 Genomes Project and obtained from the Broad Institute resource (https://alkesgroup.broadinstitute.org/LDSCORE/). Univariate LDSC was used to estimate SNP-based heritability (*h*^2^) for all traits. Only traits with statistically significant heritability (*p*-value < 0.05) were retained for downstream analyses, ensuring that MR and SMR were applied only to traits with detectable polygenic signal. The harmonized datasets were stored in a centralized database system, forming the foundation for subsequent LDSC, MR, and SMR analyses within MOCR-DB.

### Phenotype-to-Phenotype Genetic Correlation and Causality

To further investigate shared genetic architecture, bivariate LDSC was performed to estimate genetic correlations (*r*_g_) between trait pairs. Analyses were restricted to cases where the exposure and outcome originated from distinct GWAS datasets or cohort sources, where they belonged to distinct UMLS categories, and where both exhibited significant heritability. This filtering strategy enhanced the robustness and interpretability of the resulting correlation network.

All heritability and genetic correlation estimates were incorporated into MOCR-DB and were available through interactive, network-based visualizations.

In addition, a genetic correlation network was generated by representing each trait as a node and connecting trait pairs with significant genetic correlation (*p*-value < 0.05). Edge weights corresponded to the estimated correlation coefficients, and node attributes encoded heritability and cohort source. This module allows users to identify traits with robust polygenic signals and to explore cross-trait genetic architectures across populations, forming a foundational layer for causal inference in MOCR-DB.

Building on the heritability and correlation patterns identified by LDSC, MR was applied to evaluate potential causal relationships between traits. MR uses genetic variants as instrumental variables to help distinguish correlation from causation, allowing refinement of trait pairs that show genetic overlap into those with evidence of directional effects.

Four complementary MR approaches were implemented, including inverse-variance weighted (IVW) (Burgess et al. 2016), MR-Egger (Bowden et al. 2015), weighted median model (Bowden et al. 2016), and weighted mode model (Hartwig et al. 2017). These methods differ in their assumptions regarding horizontal pleiotropy, which can confound causal inference. IVW assumes balanced pleiotropy with no correlated effects, MR-Egger accounts for uncorrelated pleiotropy through an intercept, the weighted median provides robust estimates when fewer than half of the instruments are pleiotropic, and the weighted mode estimates effects from the most consistent group of variants. Instrumental variables were selected following the criteria of Zhu et al. (2018), requiring at least 10 independent genome-wide significant SNPs (*p*-value < 5 × 10^− 8^). Analyses were further limited to cases where the exposure and outcome originated from different populations, the traits belonged to distinct UMLS categories, and the exposure showed significant heritability (*p*-value < 0.05). All analyses were conducted using the TwoSampleMR package (version 0.5.5).

The MR results were incorporated into MOCR-DB and were available through searchable tables, forest plots, and scatter plots, providing users with access to causal effect estimates across traits. A directed causal network was constructed by linking exposure and outcome traits with evidence of causal association, defined as an IVW *p*-value < 0.05, consistent beta directions across MR methods, and non-significant pleiotropy and heterogeneity tests.

### Phenotype-to-Gene Module Linking Traits to Molecular Mediation

Whereas LDSC and MR address the polygenic basis and causal links of complex traits, SMR (Zhu et al. 2018) was applied to investigate whether these associations may be mediated through intermediate molecular traits. SMR integrates GWAS summary statistics with QTL datasets to evaluate whether the effect of a genetic variant on a complex trait is mediated through changes in gene expression or other molecular traits. By incorporating QTL data across diverse tissues, this framework not only links trait associations to molecular regulation but also enables exploration of tissue-specific regulatory differences, which also allows comparisons of tissue-specific associations across different biological contexts.

SMR integrates GWAS summary statistics with diverse QTL resources, including expression, methylation, splicing, and chromatin accessibility QTLs, thereby enabling systematic prioritization of gene-level functional associations. Outcomes were restricted to traits with significant heritability as determined by LDSC to ensure adequate polygenic signal for downstream analyses. In MOCR-DB, associations with *P*_SMR_ < 1 × 10^− 3^ and *P*_HEIDI_ > 0.05 were retained as candidate functional gene associations for downstream annotation and pathway-level interpretation. This threshold was used as an exploratory filtering criterion to balance signal detection and interpretability across heterogeneous GWAS traits and QTL datasets, rather than as a strict confirmatory significance cutoff. SMR-associated genes were then analyzed using Gene Ontology (GO) (Gene Ontology 2021), Kyoto Encyclopedia of Genes and Genomes (KEGG) (Kanehisa and Goto 2000), and Reactome (Gillespie et al. 2022) pathway enrichment to summarize biological processes and signaling pathways that may be related to the observed associations. Within MOCR-DB, SMR and enrichment results are presented as bar charts and searchable tables, allowing users to examine gene-level findings in the context of broader functional categories.

### Database Implementation and Interactive Visualization

Results from LDSC, MR, and SMR analyses were integrated into MOCR-DB, which was developed as an interactive web application using the R package Shiny within a modular architecture. The interface was designed to support interactive browsing, result filtering, and visualization across different analysis modules. Visualization was implemented using R libraries including networkD3 for network diagrams, plotly for statistical graphics, and DT for large-scale data tables. The SQLite database served as the backend to enable efficient synchronization between user inputs and server responses. A complete list of software dependencies used for database construction is provided in Supplementary File 1.

Interactive visualization was implemented as a central feature of MOCR-DB. Network views allow users to explore relationships between traits, with node size and color representing heritability, effect sizes, or statistical significance. Additional plots, including forest plots, scatter plots, heatmaps, and doughnut charts, provide complementary perspectives on genetic correlation and causal estimates. To enhance interpretability, MOCR-DB incorporates an AI-assisted module powered by the ModelScope API (Qwen/Qwen3.5-397B-A17B, https://api-inference.modelscope.cn/), which provides plain-language summaries and biological context for the results. This feature helps users connect statistical findings with potential biological mechanisms in a consistent and accessible manner. All outputs are linked to searchable and downloadable tables, enabling flexible inspection of trait pairs and gene-level associations. To improve usability, we added usage instructions in the About section of MOCR-DB for the Genetic Causality, Genetic Correlation, Functional Genes, and AI-assisted interpretation modules. These instructions provide brief guidance on trait selection, parameter adjustment, result visualization, table inspection, data download, and AI-assisted summary generation.

The workflow of MOCR-DB consists of three steps. First, a central trait is selected to build correlation and causality networks. Second, specific exposure-outcome pairs can be examined in more detail, including LDSC, MR, and SMR results with functional annotations. Third, an AI-assisted module provides plain-language summaries and contextual interpretation of statistical outputs, helping users to link results with potential biological mechanisms in an accessible manner.

### Case Study Design

To illustrate the application of MOCR-DB, we conducted a case study on obesity (FinnGen) and COVID-19 outcomes from the COVID-19 HGI. Two phenotypes were analyzed using the internal trait names defined in MOCR-DB: Very severe respiratory confirmed COVID-19 (‘COVID-19 Very Severe’) and hospitalized cases (‘COVID-19 Hospitalized’). For ease of interpretation, these correspond to the commonly used terms critical COVID-19 and hospitalized COVID-19, respectively. These trait pairs were evaluated using LDSC and MR to assess genetic correlation, potential causal relationships, and differences in the effect of obesity on the two COVID-19 phenotypes. In parallel, SMR was applied to contrast critical COVID-19 and hospitalized COVID-19 with lung eQTL data to identify tissue-specific molecular mediators.

In addition to trait-level SMR analyses, we further organized all SMR results from blood eQTL datasets according to standardized UMLS trait categories. SMR associations belonging to the same UMLS disease or phenotype group were aggregated to enable a systematic comparison across related traits. For each UMLS category, gene-level SMR effect estimates (*β*) were summarized into heatmaps, allowing visualization of shared and distinct molecular patterns across phenotypically related traits. This categorization provides an additional layer of interpretability by enabling comparison of effect directions and magnitudes across trait clusters.

## Results

### Overview of MOCR-DB Framework

**Fig. 1:**
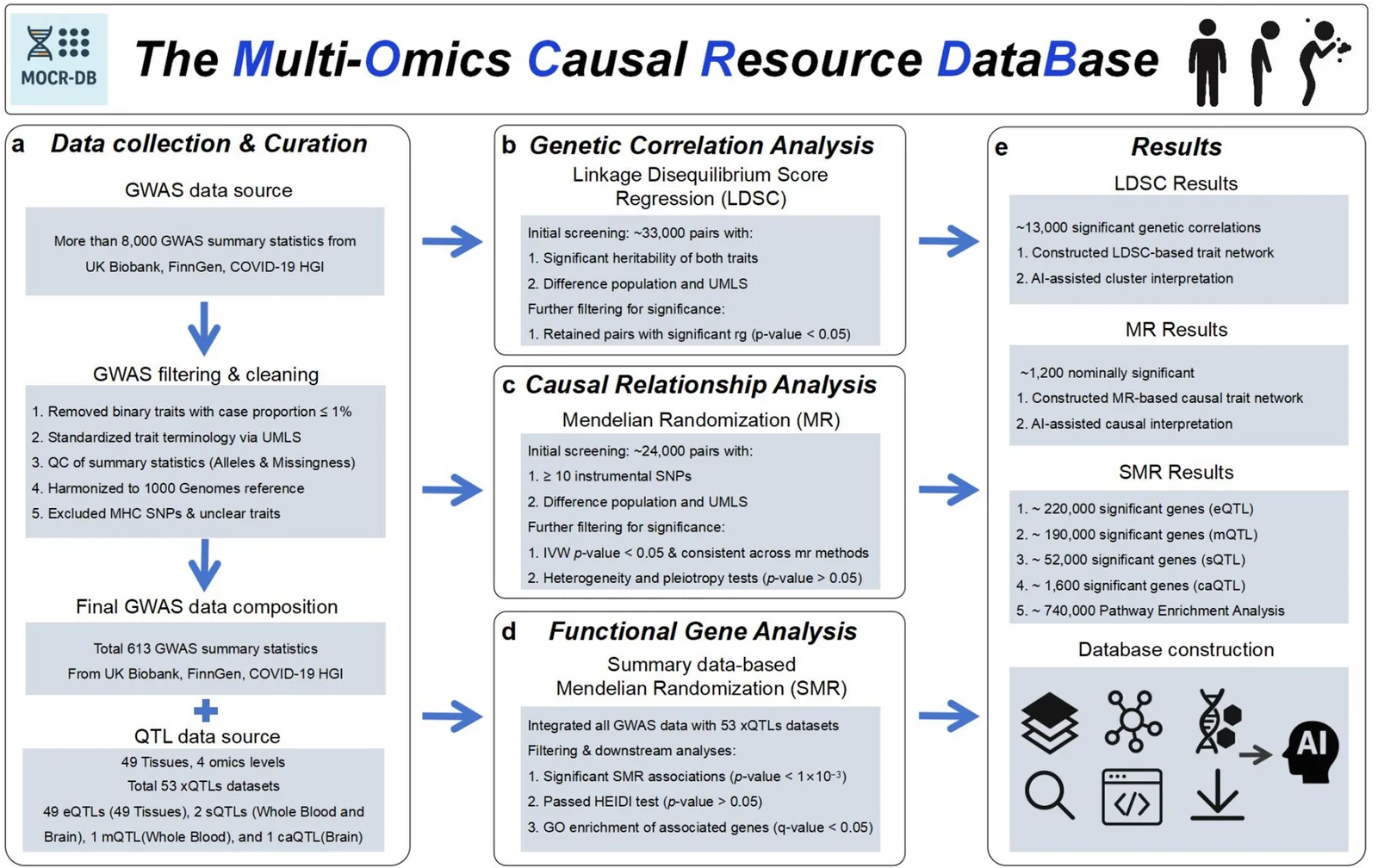
Overview of the MOCR-DB data processing and analysis framework. **a** Data collection and curation. More than 8000 GWAS summary statistics from UK Biobank, FinnGen, and the COVID-19 Host Genetics Initiative were collected, quality-controlled, standardized using UMLS, and harmonized with the 1000 Genomes reference. After removing binary traits with case proportions ≤ 1%, MHC SNPs, and traits with unclear definitions, 613 GWAS datasets were retained and integrated with 53 xQTL datasets, including 49 eQTL, 2 sQTL, 1 mQTL, and 1 caQTL datasets across 49 tissues. **b** Genetic correlation analysis. Linkage disequilibrium score regression (LDSC) was used to estimate genetic correlations between trait pairs. Pairs with significant heritability and non-overlapping cohorts sources were screened, and those with significant genetic correlation were retained for network construction. **c** Causal relationship analysis. Mendelian randomization (MR) was used to evaluate potential causal relationships between exposure and outcome traits. Pairs with adequate instrumental variables were filtered based on IVW significance, consistent effect directions across MR methods, and non-significant heterogeneity and pleiotropy tests. **d** Functional gene analysis. Summary-data-based Mendelian randomization (SMR) was used to integrate GWAS datasets with eQTL, sQTL, mQTL, and caQTL resources. Associations passing SMR and HEIDI test thresholds were retained for pathway enrichment analysis. **e** Results and database construction. The outputs included approximately 13,000 significant genetic correlations, ~ 1200 MR-supported causal trait pairs, ~ 220,000 eQTL-based SMR genes, ~ 190,000 mQTL genes, ~ 52,000 sQTL genes, ~ 1600 caQTL genes, and ~ 740,000 significant pathway enrichments. These results were organized into MOCR-DB for interactive exploration, network visualization, functional interpretation, and AI-assisted summarization

MOCR-DB (https://chenhongwei.net/public/MOCRdb/) was developed as an interactive platform for exploring genetic relationships across human traits. The overall workflow for data collection, quality control, harmonization, and downstream analyses is summarized in Fig. 1. Large-scale GWAS summary statistics were obtained from UK Biobank, FinnGen, and the COVID-19 Host Genetics Initiative, followed by standardized trait annotation, variant-level quality control, and alignment with the 1000 Genomes reference panel. Traits with unclear phenotype definitions or insufficient case counts were excluded, and the curated set of traits served as the foundation for all subsequent analyses.

**Fig. 2:**
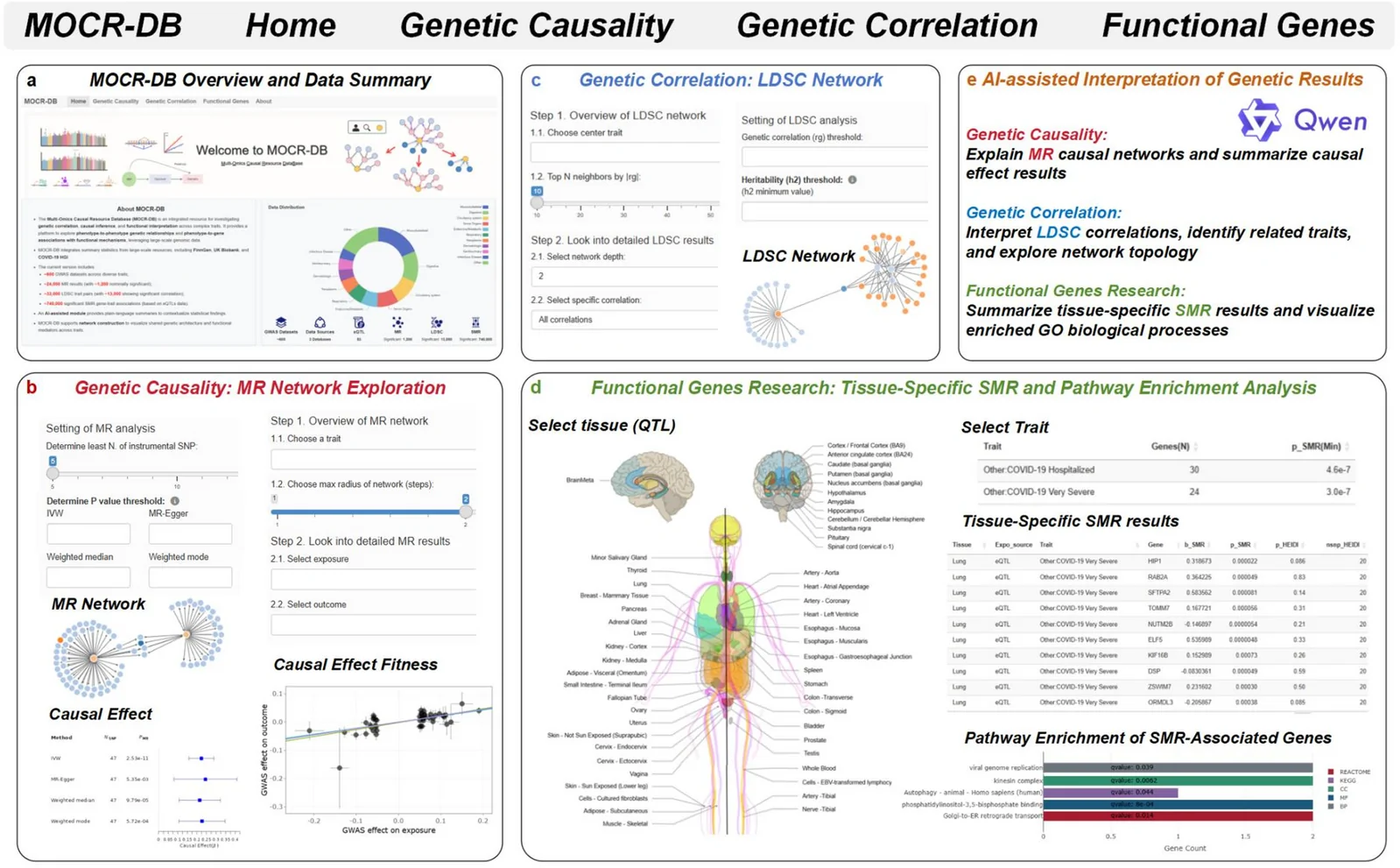
Overview of the MOCR-DB interface and core analytical modules. This figure summarizes the main interface and analytical structure of MOCR-DB. **a** The homepage provides an overview of the database, including the major data sources, trait composition, and entry points to the analytical modules. The lower panels summarize the overall trait categories and available analysis functions, allowing users to access different layers of results from a single interface. **b** The Genetic Causality module displays Mendelian randomization results in an interactive network format. Users can select a central trait, adjust the number of neighboring traits and significance thresholds, and inspect detailed causal effect estimates across multiple MR methods, including IVW, MR-Egger expression, weighted median, and weighted mode. The module also provides visual summaries of causal estimates and SNP-level effect patterns. **c** The Genetic Correlation module presents LDSC-derived genetic correlation results. Users can select a trait of interest, explore genetically related traits, and examine network structures based on genetic correlation estimates. **d** The Functional Genes module integrates tissue-specific SMR results from 53 xQTL datasets. It allows users to select QTL-derived tissue sources and traits, examine SMR-associated genes, and view pathway enrichment results for the corresponding gene sets. **e** The AI-assisted interpretation panel provides concise summaries for MR, LDSC, and SMR results. This module is intended to help users interpret statistical outputs and place genetic findings into a broader biological context. Overall, MOCR-DB combines phenotype-to-phenotype analyses, including genetic correlation and MR, with phenotype-gene analyses based on SMR within one interactive platform

The platform interface was designed to provide intuitive access to three analytical components: genetic causality (MR), genetic correlation (LDSC), and functional genomics (SMR and pathway enrichment). Figure 2 provides an overview of the interface layout, including module navigation, interactive visualization panels, and the AI-assisted summary tool. Figure 2a presents the homepage of MOCR-DB, summarizing the overall data structure, trait coverage, and entry points to each analytical module. Figure 2b shows the layout of the Genetic Causality module, which provides network-based visualization of Mendelian randomization analyses and enables users to explore directional relationships between traits. Figure 2c highlights the Genetic Correlation module, where LDSC-derived correlation matrices and network structures can be interactively examined to assess polygenic overlap. Figure 2d illustrates the Functional Genomics module, showing how SMR-derived gene-level associations and pathway enrichment results are integrated across 53 xQTL datasets. Figure 2e showcases the AI-assisted summary tool, which generates concise text interpretations to aid the biological contextualization of statistical outputs. To provide more detailed operational guidance, Supplementary Figs. S1 and S2 further illustrate the interface structure of the main analytical modules. Fig. S1 shows the MR-based Genetic Causality module and the LDSC-based Genetic Correlation module, while Fig. S2 presents the SMR-based Functional Genes module, including trait and tissue selection, result tables, enrichment visualization, and AI-assisted interpretation.

Together, these components establish MOCR-DB as a unified framework for investigating causal relationships, polygenic overlap, and molecular mediators across a broad range of human traits.

### Summary of GWAS Data

From an initial collection of more than 8000 GWAS summary statistics, 613 traits were retained after applying standardized terminology, variant-level QC, reference harmonization, and excluding traits with unclear definitions or low case proportions. These traits encompass complex diseases, quantitative clinical measurements, and two COVID-19 severity phenotypes.

Univariate LDSC was then used to estimate SNP-based heritability (*h*^*2*^) for each trait. Only traits with nominally significant heritability (*p*-value < 0.05) were included to ensure sufficient polygenic signal for downstream MR and SMR analyses. The complete list of heritable traits incorporated into MOCR-DB, together with their heritability estimates, is provided in Table S1.

### Phenotype-to-Phenotype Genetic Relationships

Potential causal relationships between traits were evaluated using multiple MR approaches. A total of 23,892 exposure-outcome trait pairs met the criteria for MR testing. After filtering by IVW *p*-value < 0.05, consistent effect directions across at least two MR methods, and non-significant heterogeneity and pleiotropy statistics, 1215 putative causal pairs were retained. The trait pairs included in the MR analyses are summarized in Table S2, and their interactive exploration is supported through the interface shown in Fig. 2b.

To complement directional causal inference, bivariate LDSC was applied to quantify cross-trait genetic correlations among the 613 heritable traits. After removing pairs from the same cohort or with overlapping UMLS categories, 32,721 trait pairs were evaluated. Among these, 13,416 pairs showed significant genetic correlation (*p*-value < 0.05), revealing extensive shared genetic architecture across complex traits. The assessed trait pairs are listed in Table S3, and the correlation visualization interface is illustrated in Fig. 2c.

### Phenotype-to-Gene Associations and Functional Mechanisms

To identify gene-level mediators underlying complex traits, SMR was performed by integrating 53 xQTL datasets (49 eQTLs, 2 sQTLs, 1 mQTL, and 1 caQTL) with the 613 heritable traits. Using the exploratory filtering criterion < 1 × 10^− 3^, the SMR analysis identified 215,276 trait-gene associations from eQTL datasets, 191,921 from mQTL datasets, 52,472 from sQTL datasets, and 1654 from the caQTL dataset. These gene-level associations form a large compendium of potential molecular mediators linking genetic variants to phenotypic variation. An overview of how these SMR results are displayed within the Functional Genomics module is shown in Fig. 2d, where users can explore gene-level effects and downstream pathway enrichment results across all integrated xQTL datasets.

To characterize the biological processes involving these SMR-associated genes, pathway enrichment analyses were performed using GO, KEGG, and Reactome databases. Across all SMR-prioritized genes, 742,573 enriched pathways (*q*-value < 0.05) were identified, reflecting diverse biological programs including immune regulation, metabolic signaling, neuronal function, and tissue-specific regulatory mechanisms. These enrichment results can be interactively examined within MOCR-DB, with the exploration interface demonstrated in Fig. 2d. To further aid interpretation, the AI-assisted summary tool (Fig. 2e) provides concise textual descriptions that accompany SMR and enrichment outputs, helping contextualize statistical findings within relevant biological processes.

### Case Study: Genetic Correlation and Causal Effects of Obesity on COVID-19 Severity

As a representative example, we examined the relationship between obesity and COVID-19 severity. LDSC indicated significant genetic correlations between obesity (FinnGen) and both critical COVID-19 and hospitalized COVID-19 (Table S5). MR analyses consistently supported a potential causal effect of obesity on both outcomes.

**Fig. 3:**
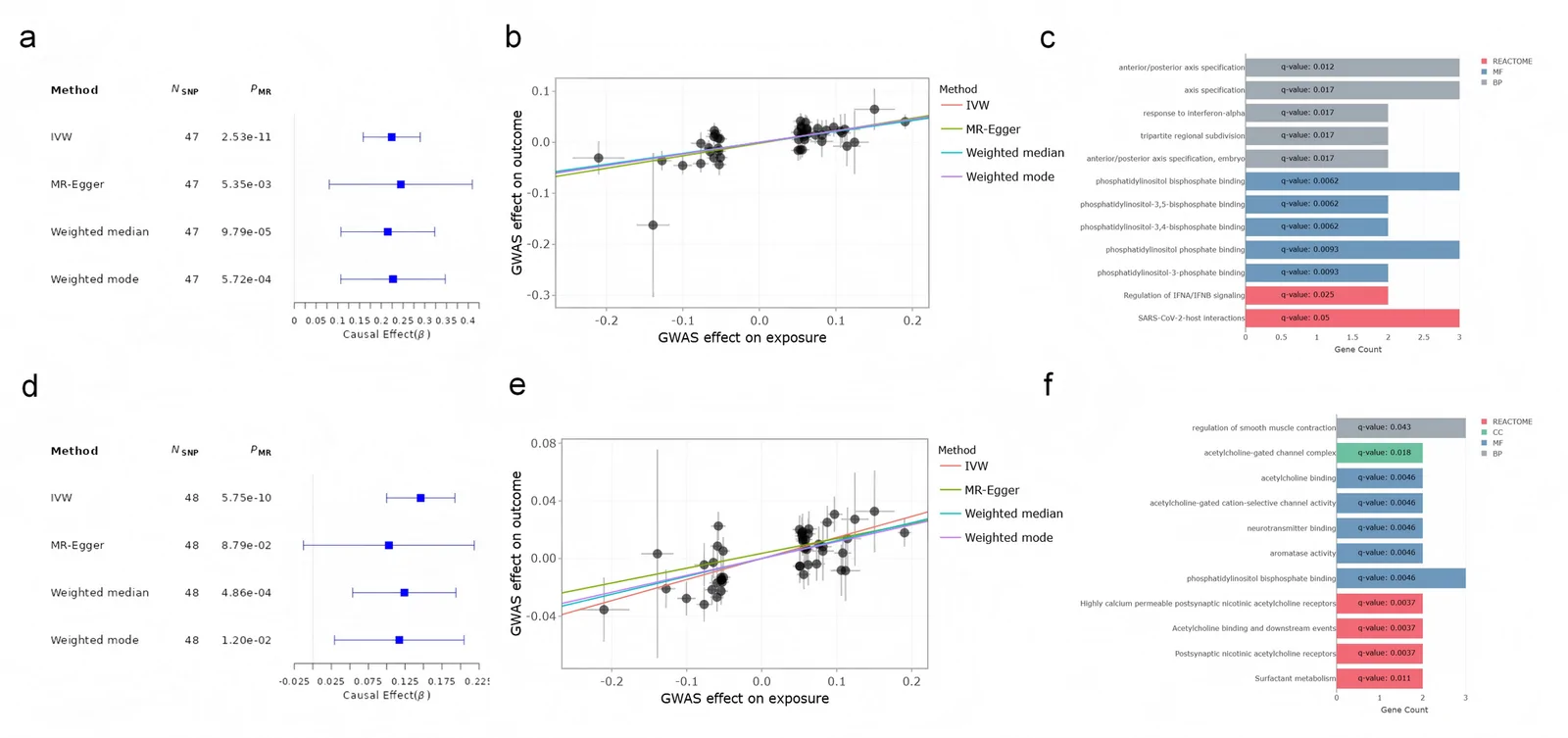
Causal effect estimates of obesity on COVID-19 severity and tissue-specific SMR signatures. This figure illustrates the use of MOCR-DB to connect phenotype-level causal analysis with tissue-specific functional gene analysis, using obesity and COVID-19 severity as an example. **a**-**b** Mendelian randomization analysis of genetically predicted obesity on critical COVID-19. Panel a shows causal effect estimates from four MR methods, including IVW, MR-Egger, weighted median, and weighted mode. The estimates were positive across the four methods, with different levels of statistical support. Panel b shows the corresponding MR scatter plot, in which each point represents a genetic instrument and the fitted lines represent estimates from different MR methods. **c** Pathway enrichment analysis of lung SMR-associated genes for critical COVID-19. The enriched terms included interferon-related immune processes, SARS-CoV-2 host interaction, and phosphatidylinositol-binding-related functions, suggesting that lung-expressed genes associated with critical COVID-19 were enriched in immune and signaling-related pathways. **d**-**e** Mendelian randomization analysis of genetically predicted obesity on hospitalized COVID-19. Panel d shows effect estimates from the same four MR methods, and Panel e presents the corresponding SNP-level scatter plot. The MR estimates were also positive, although the overall pattern was less pronounced than that observed for critical COVID-19. **f** Pathway enrichment analysis of lung SMR-associated genes for hospitalized COVID-19. The enriched terms included cholinergic synaptic processes, smooth muscle regulation, acetylcholine-related pathways, and surfactant-related pathways. Compared with Panels a-c, Panels d-f show that obesity-related MR signals were observed for both COVID-19 severity phenotypes, whereas the lung SMR-associated enrichment patterns differed between critical COVID-19 and hospitalized COVID-19

For critical COVID-19, effect estimates ranged from *β* = 0.215 to 0.245, with *p*-values spanning from 2.5 × 10^− 11^ to 5.7 × 10^− 4^. For hospitalized COVID-19, effect estimates ranged from *β* = 0.103 to 0.146, with *p*-values spanning from 5.8 × 10^− 10^ to 8.8 × 10^− 2^. Pairwise *t*-test across the four MR methods indicated significant differences in causal effect estimates (*p*-value = 4.8 × 10^− 4^). Importantly, MR-Egger produced consistent effect directions across both phenotypes, and the intercept test did not indicate directional pleiotropy. Collectively, these findings suggest that obesity exerts a stronger genetic effect on the risk of critical COVID-19, compared with hospitalized COVID-19 (Fig. 3 and Table S6).

### Case Study: Molecular Differentiation of COVID-19 Severity Based on Lung SMR and Pathway Analyses

To further dissect the observed differences between these two COVID-19 severity phenotypes, SMR analyses were conducted using lung eQTL data followed by GO, KEGG, and Reactome enrichment of SMR-prioritized genes, reflecting the primary site of COVID-19 pathology (Fig. 3). For critical COVID-19, these genes were enriched for interferon- and virus-related pathways, including response to interferon-alpha, regulation of *IFNA/IFNB* signaling, and SARS-CoV-2 host interactions, together with developmental and patterning terms such as axis specification and anterior/posterior axis specification (Table 1). Several categories related to phosphatidylinositol and phosphatidylinositol-bisphosphate binding were also enriched, suggesting a prominent role for lipid-mediated signaling in the progression to critical disease. In contrast, genes associated with hospitalized COVID-19 showed a distinct functional profile dominated by neuronal and cholinergic processes, including acetylcholine-gated channel complex, acetylcholine binding, neurotransmitter binding, and postsynaptic nicotinic acetylcholine receptors, alongside terms related to regulation of smooth muscle contraction and surfactant metabolism. Although both phenotypes shared enrichment in phosphatidylinositol-bisphosphate binding, the overall patterns indicate that critical cases are characterized by stronger antiviral and inflammatory signaling, whereas hospitalized COVID-19 are more prominently linked to cholinergic synaptic transmission and airway function. These pathway-level findings demonstrate how MOCR-DB can capture symptom-differentiating molecular signatures between clinically related phenotypes.

**Table 1:** SMR results integrating lung eQTL data with COVID-19 severity phenotypes.

Gene	Probe	CHR	β_GWAS_	P_GWAS_	β_eQTL_	P_eQTL_	β_SMR_	P_SMR_	P_HEIDI_
IFNAR2	ENSG00000159110	21	0.066	9.08E-06	0.148	1.19E-08	0.448	4.62E-04	3.46E-01
IFNAR1	ENSG00000142166	21	-0.090	5.28E-08	0.284	4.79E-23	-0.318	1.87E-06	1.28E-01
HIP1	ENSG00000127946	7	0.074	5.14E-07	0.232	1.81E-15	0.319	2.18E-05	8.56E-02
RAB2A	ENSG00000104388	8	0.066	4.02E-06	0.182	1.19E-17	0.364	4.94E-05	8.30E-01
SFTPA2	ENSG00000185303	10	0.082	1.45E-07	0.140	2.57E-09	0.584	8.06E-05	1.45E-01
TOMM7	ENSG00000196683	7	-0.063	3.53E-05	-0.378	1.74E-71	0.168	5.58E-05	3.06E-01
NUTM2B	ENSG00000188199	10	-0.106	1.40E-06	0.722	4.24E-42	-0.147	5.42E-06	2.11E-01
ELF5	ENSG00000135374	11	-0.129	3.97E-08	-0.241	1.49E-16	0.536	4.80E-06	3.25E-01
KIF16B	ENSG00000089177	20	-0.074	3.41E-04	-0.484	3.46E-24	0.153	7.30E-04	2.64E-01
DSP	ENSG00000096696	6	0.061	3.14E-05	-0.729	3.68E-75	-0.083	4.90E-05	5.92E-01
ZSWIM7	ENSG00000214941	17	0.056	7.92E-05	0.240	3.14E-19	0.232	3.04E-04	4.97E-01
ORMDL3	ENSG00000172057	17	0.052	1.48E-04	-0.254	6.66E-24	-0.206	3.84E-04	8.48E-02
GSDMA	ENSG00000167914	17	-0.060	1.48E-05	-0.806	1.10E-72	0.074	2.53E-05	1.10E-01
WNT3	ENSG00000108379	17	0.130	4.40E-09	-0.377	9.12E-26	-0.346	3.02E-07	5.18E-01
SNRPD2	ENSG00000125743	19	0.058	3.67E-04	-0.204	1.47E-22	-0.283	8.17E-04	1.98E-01
TULP2	ENSG00000104804	19	-0.068	5.04E-04	0.732	1.62E-24	-0.093	9.91E-04	5.99E-02
NAPSB	ENSG00000131401	19	0.081	2.26E-07	-0.545	7.80E-43	-0.148	1.28E-06	9.57E-02
TDRD10	ENSG00000163239	1	0.066	1.97E-04	-0.496	1.91E-45	-0.134	3.18E-04	7.53E-02
FUBP1	ENSG00000162613	1	0.111	1.17E-06	-0.258	1.52E-13	-0.431	4.91E-05	5.23E-01
SLC9A3	ENSG00000066230	5	-0.059	1.48E-04	0.791	8.24E-59	-0.075	2.21E-04	6.50E-01
RFTN2	ENSG00000162944	2	0.061	1.18E-05	-0.191	4.91E-19	-0.318	8.42E-05	3.39E-01
INTS12	ENSG00000138785	4	0.112	1.77E-05	-0.386	1.03E-12	-0.292	2.36E-04	2.11E-01
NPNT	ENSG00000168743	4	0.113	2.51E-05	-0.390	5.81E-13	-0.289	2.75E-04	6.71E-02
GSDMB	ENSG00000073605	17	-0.036	1.54E-04	0.216	1.53E-22	-0.166	4.17E-04	3.87E-01
ORMDL3	ENSG00000172057	17	0.039	3.49E-05	-0.254	6.66E-24	-0.153	1.29E-04	3.81E-01
CHRNA7	ENSG00000175344	15	0.088	1.04E-04	-0.889	2.06E-12	-0.099	6.78E-04	3.40E-01
CENPV	ENSG00000166582	17	0.038	6.03E-05	-0.274	1.89E-12	-0.139	4.91E-04	1.12E-01
GSDMA	ENSG00000167914	17	-0.037	9.83E-05	-0.806	1.10E-72	0.046	1.41E-04	1.35E-01
WNT3	ENSG00000108379	17	0.072	8.97E-09	-0.377	9.12E-26	-0.192	4.60E-07	1.03E-01
DLX3	ENSG00000064195	17	0.219	3.62E-14	0.854	2.05E-11	0.256	5.18E-07	5.61E-02
IFNAR1	ENSG00000142166	21	-0.054	2.46E-06	0.284	4.79E-23	-0.190	2.11E-05	1.66E-01
KREMEN1	ENSG00000183762	22	0.044	2.22E-05	-0.214	7.68E-10	-0.204	4.79E-04	4.62E-01
ZNF778	ENSG00000170100	16	0.067	2.12E-06	0.208	2.82E-10	0.324	1.50E-04	1.27E-01
ADORA2B	ENSG00000170425	17	0.042	6.23E-05	-0.241	8.38E-14	-0.173	4.18E-04	5.65E-02
ZSWIM7	ENSG00000214941	17	0.043	8.55E-06	0.240	3.14E-19	0.177	6.71E-05	2.16E-01
SFTPA2	ENSG00000185303	10	0.047	1.43E-05	0.140	2.57E-09	0.336	4.53E-04	9.36E-02
ELF5	ENSG00000135374	11	-0.075	9.20E-09	-0.241	1.49E-16	0.309	2.41E-06	7.73E-02
CHRFAM7A	ENSG00000166664	15	0.092	1.21E-05	-0.924	1.30E-16	-0.100	1.10E-04	4.87E-01
GOLGA8R	ENSG00000186399	15	0.091	1.64E-05	-0.814	1.03E-14	-0.112	1.67E-04	6.18E-01
OXCT2P1	ENSG00000237624	1	0.044	4.70E-04	0.718	1.31E-27	0.061	8.69E-04	8.82E-02
CYP4B1	ENSG00000142973	1	-0.049	4.77E-04	-0.662	2.50E-61	0.074	6.32E-04	7.14E-02
CYP4Z1	ENSG00000186160	1	-0.054	1.09E-04	-0.467	1.51E-17	0.115	4.25E-04	3.99E-01
FUBP1	ENSG00000162613	1	0.092	3.41E-09	-0.258	1.52E-13	-0.357	3.94E-06	4.96E-01
IBA57	ENSG00000181873	1	-0.041	3.78E-05	0.375	5.15E-16	-0.109	2.39E-04	2.59E-01
ASPRV1	ENSG00000244617	2	0.079	6.93E-05	-0.448	1.15E-09	-0.176	8.67E-04	7.43E-01
PCBP1-AS1	ENSG00000179818	2	0.072	2.32E-04	-0.442	1.53E-18	-0.164	6.86E-04	3.06E-01
INTS12	ENSG00000138785	4	0.079	1.49E-05	-0.386	1.03E-12	-0.204	2.15E-04	2.06E-01
NPNT	ENSG00000168743	4	0.081	9.83E-06	-0.390	5.81E-13	-0.208	1.64E-04	5.60E-02
SLC9A3	ENSG00000066230	5	-0.041	1.43E-04	0.791	8.24E-59	-0.051	2.14E-04	4.16E-01
TOMM7	ENSG00000196683	7	-0.039	1.70E-04	-0.378	1.74E-71	0.102	2.33E-04	7.77E-02
HIP1	ENSG00000127946	7	0.041	6.41E-05	0.232	1.81E-15	0.177	3.55E-04	1.48E-01
RAB2A	ENSG00000104388	8	0.050	3.16E-07	0.182	1.19E-17	0.276	1.14E-05	6.94E-01
DEPTOR	ENSG00000155792	8	0.036	1.85E-04	-0.280	4.02E-17	-0.127	6.35E-04	4.53E-01

While these pathway-level contrasts outline broad biological differences, gene-level SMR signals further revealed the specific molecular mediators that drive these divergent responses. Lung SMR analysis revealed several gene-level mediators that strongly differentiated critical from hospitalized COVID-19. The clearest signals involved the type I interferon receptor complex. For *IFNAR2*, critical COVID-19 showed a significant positive SMR effect (*β*_SMR_ = 0.448, *p*-value = 4.62 × 10^− 4^), indicating elevated receptor-linked antiviral signaling, whereas *IFNAR1* displayed a significant negative effect (*β*_SMR_ = − 0.318, *p*-value = 1.87 × 10^− 6^), which is consistent with disrupted interferon responsiveness. Together, these findings highlight interferon pathway dysregulation as a hallmark of life-threatening disease. Additional signals specific to critical COVID-19 included the surfactant-associated gene *SFTPA2* (*β*_SMR_ = 0.584, *p*-value = 8.06 × 10^− 5^), suggesting more pronounced alveolar dysfunction, as well as epithelial and vesicle-trafficking genes such as *HIP1* (*β*_SMR_ = 0.319, *p*-value = 2.18 × 10^− 6^) and *RAB2A* (*β*_SMR_ = 0.364, *p*-value = 4.94 × 10^− 6^), indicating intensified epithelial signaling and increased viral processing activity. In contrast, hospitalized COVID-19 showed weaker or less consistent SMR effects for these loci. For example, *IFNAR1* exhibited a reduced SMR effect size (*β*_SMR_ = − 0.190, *p*-value = 2.11 × 10^− 5^), and *SFTPA2* showed a markedly attenuated association (*β*_SMR_ = 0.336, *p*-value = 4.53 × 10^− 4^). Notably, no significant SMR signal for *IFNAR2* was observed in the hospitalized phenotype. These differences suggest that key interferon-related and surfactant-related pathways are more strongly perturbed in critical disease than in hospitalized COVID-19.

### Case Study: UMLS-Based Comparison of Infectious Disease SMR Signatures

**Fig. 4:**
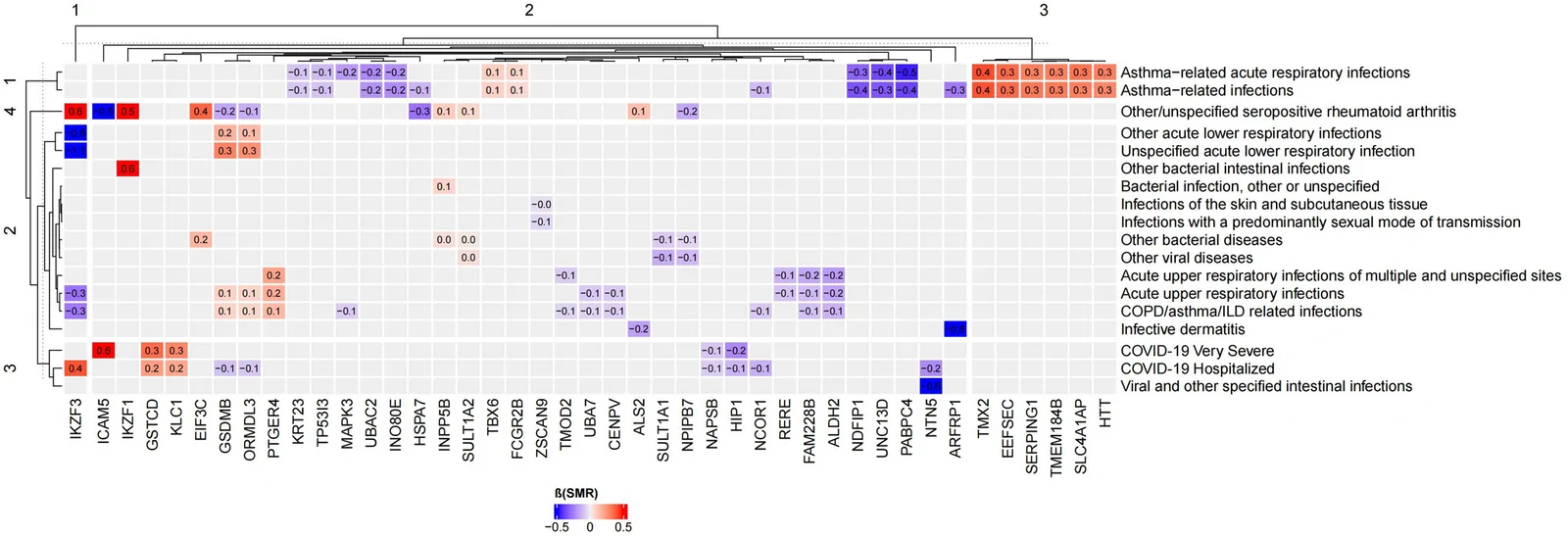
Tissue-specific SMR effect patterns across infectious disease traits grouped by UMLS categories. This heatmap summarizes gene-level SMR effect estimates for traits classified under the UMLS infectious diseases category. Rows represent infectious disease-related traits, and columns represent SMR-associated genes retained under the visualization criteria. The color scale indicates the direction and magnitude of *β*_SMR_ estimates, with red and blue representing opposite directions of genetically predicted gene-expression effects on the corresponding traits. Gray cells indicate trait-gene pairs without a displayed SMR signal under the selected criteria. Hierarchical clustering was applied to both traits and genes to visualize similarities in SMR effect patterns across infectious disease-related traits. The row clustering grouped several respiratory infection-related traits, including asthma-related infections, acute respiratory infections, and COVID-19 severity phenotypes, while the column clustering separated genes with different patterns of association across these traits. For example, very severe respiratory confirmed COVID-19 and Hospitalized COVID-19 showed partially overlapping but not identical SMR-associated gene patterns, while asthma-related respiratory infection traits formed another visually related group. These patterns suggest that MOCR-DB can be used to compare tissue-specific gene-level signals across phenotypically related diseases, rather than only retrieving single trait-gene associations

Within the infectious disease-related UMLS categories, the SMR heatmaps showed that critical COVID-19 and hospitalized COVID-19 share a distinctive molecular signature that is not seen in other respiratory or infectious phenotypes (Fig. 4).

Both COVID-19 severity traits exhibit strong and directionally consistent SMR effects in interferon-associated genes such as *IFIT2*, *IFI6*, and *OAS1*, highlighting a SARS-CoV-2-specific activation of antiviral transcriptional programs. At the same time, the two COVID-19 phenotypes also display clear within group differences, with several epithelial or membrane related genes, including *ICAM5* and other lung surface markers, showing divergent SMR effect sizes or directions between critical and hospitalized COVID-19, suggesting heterogeneous epithelial and mucosal responses across severity levels. In contrast, asthma-related infections and asthma-related acute respiratory infections present a completely different pattern. These asthma phenotypes show virtually no SMR signal in interferon pathway genes and instead are characterized by a small, coherent cluster of genes such as *TMX2*, *EEFSEC*, *SERPING1*, *TMEM184B*, *SLC4A4P*, and *HTT*, which are only weakly or not at all involved in other infectious traits. This non-interferon, asthma-specific gene set distinguishes asthma related infections from both COVID-19 and the remaining infectious disease phenotypes, indicating that asthma-associated respiratory infections are governed by distinct epithelial, complement regulatory, and mucosal pathways rather than by interferon-dominated antiviral responses.

Supplementary Fig. S3–S16 present UMLS-stratified, tissue-specific SMR heatmaps across major trait categories beyond infectious diseases. Symptoms traits (Fig. S3), Sense organs traits (Fig. S4), Self-perceived traits (Fig. S5), Respiratory traits (Fig. S6), Neurological traits (Fig. S7), Neoplasms traits (Fig. S8), Musculoskeletal traits (Fig. S9), Mental disorders traits (Fig. S10), Genitourinary traits (Fig. S11), Endocrine/Metabolic traits (Fig. S12), Digestive traits (Fig. S13), Dermatologic traits (Fig. S14), Circulatory system traits (Fig. S15), and Body Measures traits (Fig. S16) are each summarized in a dedicated panel. Within each figure, traits are grouped by UMLS categories, and tissue-specific SMR effect estimates (*β*) are visualized to facilitate category-level comparison of shared versus distinct gene-level signatures. For example, the respiratory, endocrine/metabolic, neurological, and body-measure-related panels provide additional examples of how MOCR-DB can be used to compare tissue-specific SMR patterns across clinically or biologically related traits. This organization enables users to examine directional consistency of SMR effects across tissues, identify trait clusters with coherent molecular patterns, and detect trait-specific deviations within the same high-level clinical domain. Together, these supplementary heatmaps provide a systematic atlas of UMLS-stratified, tissue-aware SMR signatures beyond infectious diseases, supporting exploratory cross-category interpretation at scale.

### AI-Assisted Analytical Summaries

MOCR-DB includes an AI-assisted summary module that provides concise summaries of MR, LDSC, SMR-prioritized genes, and downstream GO, KEGG, and Reactome enrichment results. The MR assistant offers brief descriptions of common exposure-outcome patterns observed in the causal network. The LDSC assistant gives short notes on notable genetic correlations between traits. The gene enrichment assistant summarizes the main Gene Ontology categories (BP, MF, and CC), KEGG pathways, and Reactome pathways among SMR-prioritized genes. At its core, it is an interactive hypothesis-generation aid that combines two capabilities: (1) a natural-language-to-structured query language engine that parses user's free-form questions (e.g., 'Summarize the key findings with biological insights from term descriptions') into executable database queries to fetch relevant enrichment results; and (2) the reasoning and synthesis ability of a large language model, which contextualizes the retrieved data points within its broad biomedical knowledge base to suggest interpretations, point out consistencies or contradictions with known biology, and propose potential underlying mechanisms.

## Discussion

MOCR-DB provides an integrated framework that connects phenotype-level variation with underlying genetic mechanisms, linking polygenic signals and cross-trait relationships with directional estimates and gene-level evidence. After harmonization and quality control, analyses were performed on 613 traits with significant SNP-based heritability. The platform connects statistical outputs with interpretable summaries and interactive visualizations, and incorporates an AI-assisted module that provides concise explanations to support interpretation. Compared with resources such as DMRdb (Zheng et al. 2025) or SMR-Portal (Guo et al. 2025), each of which addressed specific analytical tasks, MOCR-DB systematically bridges cross-trait overlap, causal inference, and molecular mediation within one accessible platform.

An advantage of this design is that results can be examined in a structured sequence. Cross-trait overlap is first quantified, potential causal directions are then evaluated with multiple MR approaches, and gene-level mediation is subsequently explored by integrating QTL resources across tissues. This framework was illustrated by examining obesity and COVID-19 severity phenotypes. MR analyses indicated that obesity is associated with an increased risk of both critical and hospitalized COVID-19 (Popkin et al. 2020), with stronger effects observed for the critical phenotype. Lung eQTL-based SMR was applied to compare the two severity groups. This analysis revealed distinct functional signatures: interferon-related immune and stress-response pathways were enriched in critical COVID-19 (Hadjadj et al. 2020), whereas neurotransmission and ion-channel processes were more apparent in hospitalized COVID-19. Both phenotypes also showed partial overlap in muscle-contraction and signaling pathways.

Beyond these pathway-level differences, the gene-level SMR results further demonstrated how the platform can resolve molecular mediators associated with disease severity. Within the type I interferon receptor complex, *IFNAR2* showed a positive SMR association in critical COVID-19, whereas *IFNAR1* showed a negative association, suggesting a differential disturbance of interferon responsiveness rather than a uniform shift in signaling (Arunachalam et al. 2020; Bastard et al. 2020; Hadjadj et al. 2020). Signals at *SFTPA2* (Delorey et al. 2021), together with epithelial and vesicle-trafficking genes such as *HIP1* and *RAB2A*, also pointed to stronger perturbation of surfactant function and epithelial stress in critical disease (Gordon et al. 2020). In addition, several epithelial or membrane-associated genes, including *ICAM5* (Melms et al. 2021), exhibited divergent SMR effect sizes between the two severity phenotypes, highlighting transcriptional heterogeneity in mucosal or epithelial responses that is not captured by interferon-related pathways alone (Chen et al. 2025). These findings therefore extend established knowledge by revealing additional layers of severity-specific molecular differentiation beyond canonical interferon activation.

These findings are in line with previous reports that obesity is a risk factor for critical COVID-19 (Simonnet et al. 2020; Stefan et al. 2021), and that critical COVID-19 is characterized by heightened interferon activity (Bastard et al. 2020; Zhang et al. 2020) and dysregulated immune responses in the lung (Delorey et al. 2021; Melms et al. 2021; Wang et al. 2021). At the same time, the observed epithelial and surfactant-related differences, including the divergence of *ICAM5* signals between critical and hospitalized COVID-19, demonstrate that MOCR-DB can capture both known molecular patterns and additional transcriptomic variations that may contribute to disease.

The consistency between these findings and established knowledge supports the validity of the database and illustrates the suitability of this case study. Together, these analyses show that severity-specific molecular features can be identified alongside population-level associations. These patterns were also highlighted by the AI-assisted interpretation module, which provided concise summaries that facilitated biological interpretation.

More broadly, this case study illustrates how MOCR-DB may support exploratory disease-mechanism research by connecting disease phenotypes with genetically related traits and candidate functional genes. For clinical researchers, its value may further increase as more biomarker-related GWAS traits are incorporated in future updates, including hematological indices, biochemical measurements, inflammatory markers, circulating proteins, metabolites, and immune-cell traits. Such expansion would help users examine genetic links between diseases and clinically measurable traits and prioritize candidate genes or pathways for follow-up validation.

The present version of MOCR-DB has limitations. All GWAS data included are of European ancestry, which restricts generalization to other populations. For COVID-19, only two severity phenotypes, critical and hospitalized COVID-19, were available, limiting finer stratification. Although 53 QTL resources have been integrated, proteomic and cell-type-resolved datasets remain underrepresented. In addition, the current platform relies on precomputed analyses, and user-defined uploads are not yet supported. Future work will extend the resource to other populations, incorporate additional molecular datasets such as pQTL, cell-type eQTL and open chromatin maps, and implement advanced procedures including colocalization, fine mapping and multivariable MR to strengthen causal inference. With improved computational infrastructure, controlled data submission and reproducible pipelines will also be implemented.

## Conclusion

MOCR-DB provides a unified and interactive resource for investigating shared genetic architecture, putative causal relationships, and tissue-specific molecular mediators across complex human traits. By integrating GWAS summary statistics, molecular QTL datasets, LDSC, MR, SMR, pathway enrichment, and AI-assisted interpretation within one framework, MOCR-DB supports the generation of reproducible and biologically interpretable hypotheses. The current version focuses on European-ancestry GWAS data and precomputed analyses, but it provides a scalable foundation for future expansion to broader populations, additional omics layers, and refined causal inference methods.

## Supplementary Information

Below is the link to the electronic supplementary material.

Supplementary Material 1

Supplementary Material 2

## Data Availability

All GWAS and QTL summary statistics were obtained from publicly accessible repositories. The FinnGen summary statistics were obtained from the FinnGen data portal (https://www.finngen.fi/); the UK Biobank GWAS summary statistics were obtained from the GCTA data portal (https://yanglab.westlake.edu.cn/software/gcta/); and the COVID-19 summary statistics were obtained from the COVID-19 Host Genetics Initiative release 7 platform (https://www.covid19hg.org/results/r7/). The QTL datasets used for SMR analyses, including eQTL, sQTL, mQTL, and caQTL resources, are summarized in Supplementary Table S4 with their source, version or genome build where available, access URL, and original reference.

## Abbreviations

AI: Artificial intelligence
caQTL: Chromatin accessibility quantitative trait locus
COVID-19 HGI: COVID-19 Host Genetics Initiative
eQTL: Expression quantitative trait locus
GO: Gene Ontology
GWAS: Genome-wide association study
HEIDI: Heterogeneity in dependent instruments
IVW: Inverse-variance weighted
KEGG: Kyoto Encyclopedia of Genes and Genomes
LD: Linkage disequilibrium
LDSC: Linkage disequilibrium score regression
MHC: Major histocompatibility complex
MR: Mendelian randomization
mQTL: methylation quantitative trait locus
QTL: Quantitative trait locus
SMR: Summary-data-based Mendelian randomization
SNP: Single nucleotide polymorphism
sQTL: Splicing quantitative trait locus
UMLS: Unified Medical Language System
UKB: UK Biobank
1KGP: 1000 Genomes Project

## Declarations

### Use of AI Tools

MOCR-DB includes an AI-assisted module that provides plain-language summaries and biological contextualization of precomputed LDSC, MR, SMR, and enrichment results. The module was used only as an auxiliary interpretation tool and was not involved in data generation, core statistical analyses, or the final scientific conclusions. All outputs and manuscript content were reviewed and approved by the authors.
