# Supplementary material for: MOCR-DB: The Multi-Omics Causal Resource Database for Genetic Correlation, Causal Inference, and Functional Interpretation
Source: Phenomics. 2026 Aug 28;6(5):560–74. doi: 10.1007/s43657-026-00338-w (PMC13743639; doi:10.1007/s43657-026-00338-w)
Supplement: Supplementary file 1 [file 43657_2026_338_MOESM1_ESM.docx]

**Fig. S1 Interactive interface of MOCR-DB for Genetic causality and genetic correlation analyses**

(a)The MR module of MOCR-DB. The interface is divided into a setting panel (left), a figure panel (top right), and a table panel (bottom right). Users can explore causal networks, effect size fitting, and pleiotropy tests, with an AI-assisted tool providing plain-language interpretation. (b) The LDSC module of MOCR-DB. The setting panel (left) configures heritability and correlation thresholds, while the figure panel (right) displays genetic correlation networks and the table panel lists detailed results. Download options and AI-assisted interpretation are also available.

**Fig. S2 Interactive interface of MOCR-DB for functional gene analysis using SMR**

The SMR module of MOCR-DB integrates GWAS with molecular QTL data to identify tissue-specific functional genes. The setting panel (top) allows users to select tissues and traits for analysis. The table panel (bottom left) lists significant gene-trait associations across tissues, with options to download results. The figure panel (top right) presents functional enrichment results from GO, KEGG, and Reactome analyses, with GO categories shown as an example in this interface screenshot. An AI-assisted interpretation tool (bottom right) provides plain-language summaries and interactive explanations of enriched pathways and associated biological processes.

**Fig. S3 Tissue-specific SMR effect patterns across Symptoms traits grouped by UMLS categories**

**Fig. S4 Tissue-specific SMR effect patterns across Sense Organs traits grouped by UMLS categories**

**Fig. S5 Tissue-specific SMR effect patterns across Self-perceived traits grouped by UMLS categories**

**Fig. S6 Tissue-specific SMR effect patterns across Respiratory traits grouped by UMLS categories**

**Fig. S7 Tissue-specific SMR effect patterns across Neurological traits grouped by UMLS categories**

**Fig. S8 Tissue-specific SMR effect patterns across Neoplasms traits grouped by UMLS categories**

**Fig. S9 Tissue-specific SMR effect patterns across Musculoskeletal traits grouped by UMLS categories**

**Fig. S10 Tissue-specific SMR effect patterns across Mental disorder traits grouped by UMLS categories**

**Fig. S11 Tissue-specific SMR effect patterns across Genitourinary traits grouped by UMLS categories**

**Fig. S12 Tissue-specific SMR effect patterns across Endocrine/Metabolic traits grouped by UMLS categories**

**Fig. S13 Tissue-specific SMR effect patterns across Digestive traits grouped by UMLS categories**

**Fig. S14 Tissue-specific SMR effect patterns across Dermatologic traits grouped by UMLS categories**

**Fig. S15 Tissue-specific SMR effect patterns across Circulatory system traits grouped by UMLS categories**

**Fig. S16 Tissue-specific SMR effect patterns across Body Measures traits grouped by UMLS categories**
